# State of the art of current 3-D scoliosis classifications: a systematic review from a clinical perspective

**DOI:** 10.1186/s12984-015-0083-8

**Published:** 2015-10-16

**Authors:** Sabrina Donzelli, Salvatore Poma, Luca Balzarini, Alberto Borboni, Stefano Respizzi, Jorge Hugo Villafane, Fabio Zaina, Stefano Negrini

**Affiliations:** Clinical and Experimental Sciences Department, University of Brescia, Brescia, Italy; IRCCS Fondazione Don Gnocchi, Milan, Italy; Physical and Rehabilitation Medicine Specialty, University of Milan, Milan, Italy; ISICO (Italian Scientific Spine Institute), Via Roberto Bellarmino 13/1, 20141 Milan, Italy; IRCCS Humanitas, Milan, Italy; Mechanical and Industrial Engineering Department, University of Brescia, Brescia, Italy

**Keywords:** Spine disorders, Scoliosis deformity, 3D classification, Review, Imaging

## Abstract

Scoliosis is a complex three dimensional (3D) deformity: the current lack of a 3D classification could hide something fundamental for scoliosis prognosis and treatment. A clear picture of the actually existing 3D classifications lacks. The aim of this systematic review was to identify all the 3D classification systems proposed until now in the literature with the aim to identify similarities and differences mainly in a clinical perspective.

After a MEDLINE Data Base review, done in November 2013 using the search terms “Scoliosis/classification” [Mesh] and “scoliosis/classification and Imaging, three dimensional” [Mesh], 8 papers were included with a total of 1164 scoliosis patients, 23 hyperkyphosis and 25 controls, aged between 8 and 20 years, with curves from 10° to 81° Cobb, and various curve patterns. Six studies looked at the whole 3D spine and found classificatory parameters according to planes, angles and rotations, including: Plane of Maximal Curvature (PMC), Best Fit Plane, Cobb angles in bodily plane and PMC, Axial rotation of the apical vertebra and of the PMC, and geometric 3D torsion. Two studies used the regional (spinal) Top View of the spine and found classificatory parameters according to its geometrical properties (area, direction and barycenter) including: Ratio of the frontal and the sagittal size, Phase, Directions (total, thoracic and lumbar), and Shift. It was possible to find similarities among 10 out of the 16 the sub-groups identified by different authors with different methods in different populations.

In summation, the state of the art of 3D classification systems include 8 studies which showed some comparability, even though of low level. The most useful one in clinical everyday practice, is far from being defined. More than 20 years passed since the definition of the third dimension of the scoliosis deformity, now the time has come for clinicians and bioengineers to start some real clinical application, and develop means to make this approach an everyday tool.

## Introduction

The first proposed classification for scoliosis was based on the localization of the curves according to the curve’s apex vertebra, and has been initially developed by Schulthess [1]. Then, Ponseti and Friedman revised this classification, with the approval of the Scoliosis Research Society committee [2]. They divided cases into single-curve, double-curve, and triple-curve patterns. They suggested that curve type and localization correlate with the natural history and that the curve pattern rarely changes as the spine grows. With the years, mainly for surgical purposes, two other main classifications have been developed by King et al., in 1983 [3, 4], and Lenke et al. in 2001 [5–8]. The King Classification, which allows the description of 5 types of curves, is mainly devoted to the thoracic curves, and leaves out the sagittal profile. This classification takes into consideration the curves’ type, curves’ magnitude and the degree of flexibility of the scoliosis deformity. It is simple and feasible, but has a relatively low intra-and inter-observer reliability [9]. Lenke’s classification is far more complex, being an advancement of King’s one and including lumbar and sagittal modifiers too, which represents an attempt to a more global overview of the spine. The bi-dimensionality of this widely used classification can represent a great limit if we consider the new developed technologies. New technologies offer to clinicians the opportunity to collect and automatically measure, more data related to the third dimension of the spine: the top view of the spine, the intervertebral rotation of each segment of the spine, differences in vertebral wedging, the torsion at the maximum curvature point and other [10]. Considering that scoliosis is a three dimensional deformity, a three dimensional clinical and diagnostic approach is preferable. In fact, these new measures, concerning the third dimension, can hide important risk factors. Recently, efforts to face the third dimension have been done, mainly for surgical purposes, with the aim to introduce new three-dimensional classifications systems [11–25]. In addition, SRS 3D Scoliosis Committee has recognized the need to develop a valid and clinically useful 3D classification of AIS.

An easy and quick system of classification of spine disorders, enables a better assessment of the deformity and its correlated risks, therefore, it should serve as a guide for patients’ management and also as a foundation for evidence based care. It is known that hypokyphosis as well as rotation may be associated with risk of progression, and less response to treatment. In a scoliosis with kyphosis, the apical vertebra rotation is in a sense opposite to that of the rotation of the plane of maximum curvature, and the amount of rotation of the plane of maximum curvature is greatest if the kyphosis is of lesser magnitude. A recent clinical study indicated that 3-D spinal morphology can be predictive of deformity progression [26]. The multiplicity of risk factors, and the complexity of scoliosis development point out the importance of tailored treatment, personalized according to the characteristics of each patient [27]. The main purpose of this systematic review is to identify all the three dimensional classification systems proposed until now in the scientific literature with the aim to identify the most simple to use in the everyday clinical activity and eventually develop and propose with further studies a new classification system.

## Review

### Materials and methods

This is a systematic review of all the studies presenting a three dimensional classification of scoliosis during growth published in the literature until now.

The literature was reviewed in reference to 3D scoliosis classification systems for adolescent idiopathic scoliosis. The literature pertaining to 3D classification of scoliosis was reviewed with a MEDLINE search in November 2013 using the search terms “Scoliosis/classification” [Mesh] and “scoliosis/classification and Imaging, three dimensional” [Mesh].

Two review authors independently screened the search results by reading titles and abstracts. Potentially relevant studies were obtained in full text and independently assessed for inclusion by 2 review authors, who resolved any disagreement through discussion. A third review author was contacted if disagreements persisted.

This list was narrowed using the following inclusion criteria: studies presenting comprehensive 3D classification schemes for adolescent deformity and English language.

All the studies included concerned AIS patients (in agreement with SRS criteria for diagnosis) while adults’ studies were excluded from the review process. We excluded studies in which patients presented with any type of secondary scoliosis (congenital, neurological, metabolic, post-traumatic, etc.), diagnosed according to the SRS criteria. So all scoliosis classifications analysed, regarded still in growth subjects with AIS.

Studies were included into the review independently from the type of curves or curve’s magnitude considered for the proposed three dimensional classification. The methodology of classification obtained by automatic clustering method was included with all studies using an arbitrary assignment made by scoliosis experts. All techniques used to develop 3D imaging were considered into the review.

Due to the lack of a specific quality evaluation instrument, in this review it was not carried out a methodological quality appraisal of the reviewed articles.

## Results

**Fig. 1:**
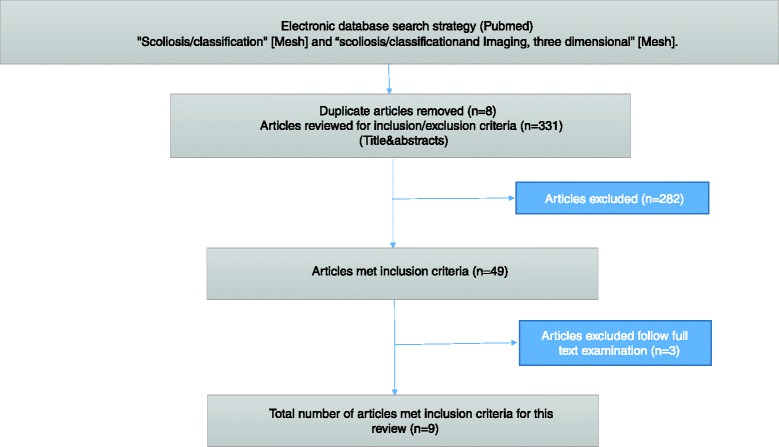
Flow chart of the systematic review performed

The search yielded 331 papers; after reviewing the titles, 49 were selected and 28 considered of interest; looking at the abstracts 14 were maintained and retrieved in full text [11–25] (Fig. 1). Subsequent assessment of the full text revealed six articles that did not met the inclusion criteria [11–14] a classification proposed by Berthonnaud et al. [12] focused on scoliotic adult patients; 3 studies didn’t propose a new classification [11, 14, 15]; two did not develop a 3D modelling of the spine [13, 24]. In the final review we included 8 studies proposing a 3D classification system for patients with a diagnosis of AIS.

The study finally included 8 papers, considering 1164 scoliosis patients, 23 hyperkyphosis and 25 controls, aged between 8 and 20 years (Table 1); only two studies provided information on gender of participants [17, 20], being 75.9 % females. The curve magnitude ranged from 10° to 81° Cobb, and curve patterns were sparse: three studies considered all curve patterns [16, 19, 20], and 3 only Lenke type 1 curves [18, 21, 23]; Stokes excluded single curves [22], Kohashi excluded single lumbar and thoracolumbar curves [17], Negrini [20] included also 23 patients with hyperkyphosis as a control group.

**Table 1:** Characteristics of included studies

Author	Patients and diagnosis			Age		Classification	COBB degrees		Design
	Idiopathic scoliosis	Normals	Notes	Average ± SD	Range		Average ± SD	Range	
DUONG	409				10-18	All		40°-	Prospective
DUONG 2	68				12-18	Lenke 1	47,2 ± 10,1°		Prospective
KADOURY	170			14 ± 2		Lenke 1 right	44±13°	11-76°	Cross sectional
KOHASHI	51				6-15	Single Thoracic; Double thoracic-lumbar		11-63°	Prospective
NEGRINI	122	20	23 hyperkyphosis	16,3 ± 2,8	12-20	All		10-55°	Cross sectional
PONCET	62	S		14	8 18	All	40°	10-71°	Cross sectional
SANGOLE	172			15 ± 2		Lenke 1 right	43±14°	10-76°	Cross sectional
STOKES	110					Double curves		9-81°	Cross sectional
Total	1164	25	23		6-20			9-81°	

Imaging systems have been used to reconstruct specific anatomic landmarks and three-dimensional coordinates in reference systems were obtained in all studies (Table 2). All the studies apart one involved a 3D reconstruction of the shape of the spine and the pelvis obtained from stereoscopic AP and LL standing radiographs. Negrini et al. [20] obtained the 3D reconstructions with an optoelectronic system (AUSCAN), an automatic device specifically developed for the postural and functional analysis of spinal deformities patients: the system compute in real-time the three-dimensional co-ordinates of a series of markers previously positioned on the skin of the analysed subject.

**Table 2:** Methodology of included studies

3D Classification system	Author	Instrument	Classification methodology	Classificatory parameters						
Full 3D				PA °C	Sag °C	PMC °C	PMC Rot	AV Rot	GT	BFP
	DUONG	Stereoradiograhies	Automatic clustering	X	X	X				
	DUONG 2	Stereoradiograhies	Automatic clustering				X		X	X
	KADOURY	Stereoradiograhies	Automatic clustering	X	X		X	X		
	PONCET	Stereoradiograhies	Qualitative visual analysis						X	
	SANGOLE	Stereoradiograhies	Automatic clustering	X	X		X	X		
	STOKES	Stereoradiograhies	Cluster analysis	X			X	X		
Spinal Tap View				Area	PA Rot	Barycenter				
	KOHASHI	Stereoradiograhies	Qualitative visual analysis	F/S Ratio	CV					
	NEGRINI	AUSCAN	Qualitative visual analysis	Phase	Total	Shift				
°C	Cobb angle									
AV	Apical Vertebra									
BFP	Best Fit Plane									
CV	Curve vectors									
F/S	Frontal/Saqittal									
GT	Geometric Torsion									
PA	Postero-Anterior									
PMC	Plane of maximum curvature									
Rot	Rotation									
Sag	Sagittal									

Some studies used clustering technique to produce the classification proposed [17, 18, 21–23] while others used an arbitrary choice made by skilled specialists (Table 2). Clustering techniques are statistical tools typically used to estimate iteratively the regrouping of data samples in a high dimensional space according to several observations or features with the hypothesis that it can define a clinically relevant classification system using the grouping of samples with similar features characterizing similar 3D curve patterns. Alternatively, the classification was developed through qualitative visual analysis of the spinal either top view [17, 20] or geometric torsion [19].

### Classificatory parameters

**Fig. 2:**
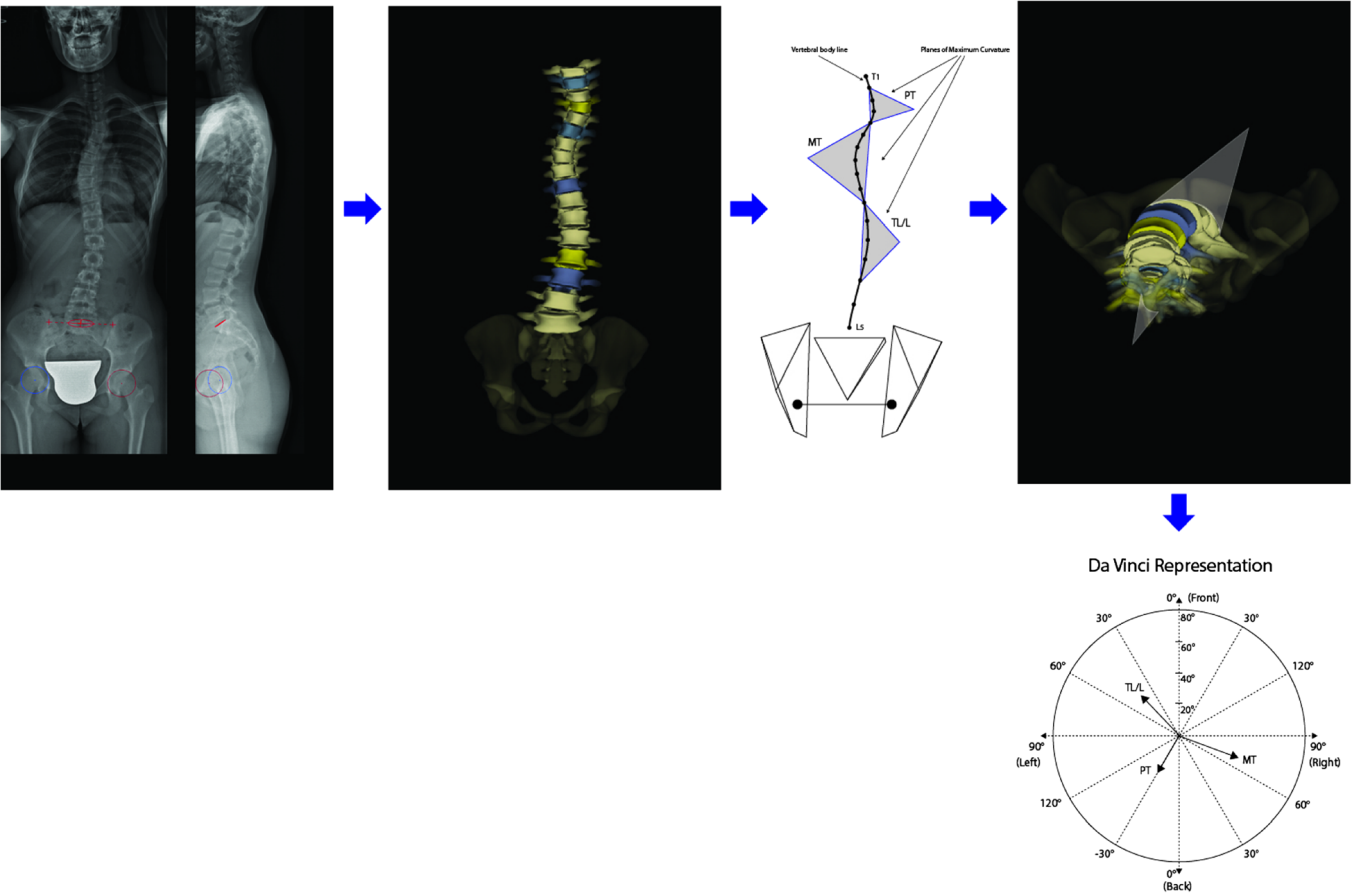
The “Plane of maximal curvature” is described by the end and apex vertebrae of each curve. The SRS committee introduced a schematic representation of the scoliotic spine called the “da Vinci representation”, which illustrates the orientation of the planes of maximum curvature of the segments in the transverse view
- Best Fit Plane (BFP) [10] (Fig. 3) is defined as the plane which minimizes the distances between the curve defined by the centroid of each vertebral body of a specified region of the spine.

In all studies the reconstructed models of the spines were used to compute 3D geometric curves and/or indices, considered as defining the shape of that patient’s spine in 3D space. Two studies focused on the spinal top view 17, 20) as the most significant and understandable description of the three-dimensional deformity, while all the others looked at the whole 3D spine [16, 18, 19, 21–23]. These last papers used classificatory parameters based on:

- Planes:
- Plane of maximal curvature (PMC) (Fig. 2), that is the plane described by the end and apex vertebrae; its orientation is computed by the curvature with respect to the sagittal plane [10, 15] in a symmetrical spine PMC lies in the sagittal plane. In presence of scoliosis, its orientation represents a composition of the coronal plane deformity and of the sagittal plane physiologic kyphosis, which may not always correspond to the projections in the sagittal and coronal planes. The SRS committee introduced a schematic representation of the scoliotic spine called the “**da Vinci representation**” (Fig. 2) that illustrates the orientation of the planes of maximum curvature of the segments in the transverse view [19].

**Fig. 3:**
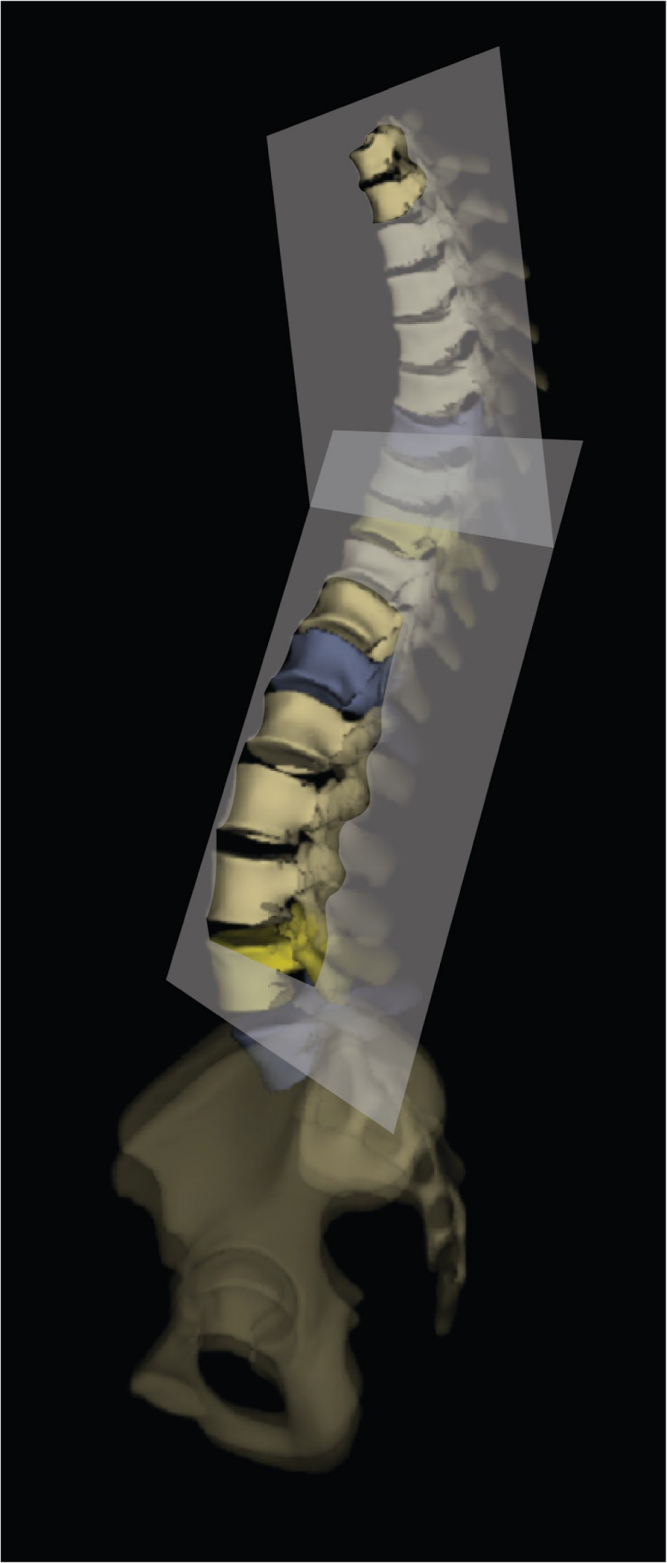
The “Best Fit Plane” (BFP) is the plane which minimizes the distances between the curve defined by the centroid of each vertebral body of a specified region of the spine
- Angles
- Classic Cobb angles of each curve in the bodily frontal and sagittal planes;
- Cobb angles in PMC [15].
- Rotations:
- Axial rotation of the apical vertebra, measured by the Stokes method [28, 29] rotation of the PMC
- Geometric torsion [19]: a true 3D measurement defined as a local geometric property of the 3D curved line passing through thoracic and lumbar vertebrae that measures the amount of helicoidal deviation of the vertebrae, without deformation of the vertebrae themselves.

**Fig. 4:**
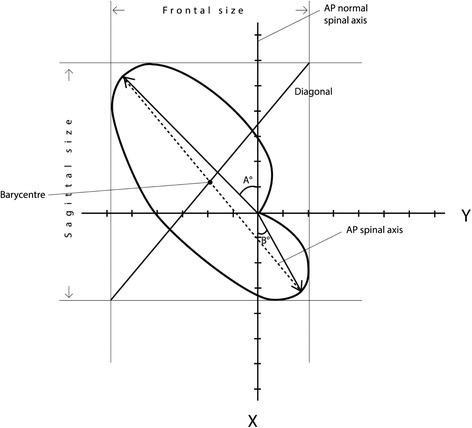
Regional spinal top view parameters as defined by Kohashi^23^ and Negrini.^26^ They identified the “Ratio of the frontal and the sagittal size” of the Top Vies, the “Phase” (obtained dividing the Top View area for the diagonal of the minimum rectangle in which the Top View is inscribable), the “Direction” (angle between the AP pathological and the AP normal spinal axes), the “Direction of the thoracic and lumbar vectors” (vectors alpha and beta, describing the maximum curvature in the thoracic and lumbar segments), the “Shift” (the displacement of the barycentre of the Top View with respect to the spinal normal vertical axis)

Kohashi [17] and Negrini [20] used the regional (spinal) top view of the spine (Fig. 4); they described geometrical parameters of the top view to classify patients as follows:

- Related to the area of the top view:
- Ratio of the frontal and the sagittal size [17]: the scoliotic angle becomes large and scoliotic deformity becomes flat on the sagittal surface when the ratio of the frontal size (deviation on the anterior and posterior surfaces) and the sagittal size (deviation on the lateral surface) are smaller than one.
- Phase [20]: is obtained dividing the Top View area for the diagonal of the minimum rectangle in which the Top View is inscribable; it is a measure of the 3D spatial evolution of the curve; this feature was defined as Phase because it takes into account the reciprocal relationship (localization and morphology) among spinal curves projected in the frontal and sagittal planes, and usually visualized at the radiographic examination; the pathological spine has new curves in the frontal plane, that may or may not be “in phase” with the physiological curves in the sagittal plane.
- Related to the postero-anterior direction of the top view:
- Overall Direction [20]: is the angle between the AP pathological spinal axis and the AP normal spinal axis; it is as if the pathological spine had changed its normal postero-anterior direction with respect to the pelvis, rotating clockwise or counter-clockwise.
- Direction of the 2 vectors describing the maximum curvature in the thoracic and lumbar segments [17]: the vectors from the center to the farthermost points of each curve from the spinal axis have a magnitude and can be balanced or not.
- Related to the barycenter (center of mass) of the top view:
- Shift [20]: is the displacement of the barycenter of the Top View with respect to the spinal normal vertical axis; it is as if the pathological spine had changed its position with respect to the pelvis, “shifting” away from the vertical C7-S1 axis.

### Subgroupings

All authors proposed subgroups (SG) according to the specific methodology followed in their studies. Some studies could not be compared, since they used either a different methodology from the others [17, 20] or did not report results similarly to other papers [19, 23]. The other papers have been compared in Table 3, and some similarities could be found among some SG, specifically:

**Table 3:** Subgroups of included studies paired for similar characteristics

Pairing	Author	Subgroups		Numerical values									
		Number	%	°C		K		L		PMC Rot		AV Rot	
				Av	SD	Av	SD	Av	SD	Av	SD	Av	SD
Paired	DUONG	5	20,0 %	42	8	23	11	30	14				
subgroups	KADOURY	2	32,4 %	59	9	26	12	-32	15	71	31	-11	3
	DUONG	1	22,7 %	43	10	25	15	38	17				
	KAOOtJRY	1	21,8 %	53	11	31	15	-39	12	61	30	-23	11
	DUONG	3	21.5 %	41	13	29	13	33	14				
	KADOURY	4	33,5 %	45	9	39	12	-33	12	S3	25	-22	S
	SANGOLE	i	12,8 %	22.4	10	35.9	6			38.2	28	-5.7	8
	STOKES	i	60,4 %	25.9	14.0					57.D	19.6	5.0	7.0
	SANGOLE	3	41,3 %	40.7	14	16.8	3			90.2	14	10.9	9
	KADOURY	3	12,4 %	45	14	19	12	-38	12	45	24	-16	8
Unpairedsubgroup;	DUONG	2	13.7 %	26	17	31	11	39	15				
	DUONG	4	22,0 %	44	11	22	13	36	12				
	SANGOLE	2	45,9 %	51.3	10	33.0	9			73.3	S	14.S	10
	STOKES	2	19.6 %	33.5	115.1					71 7	17.6	6-7	6.8
	STOKES	3	13,9 %	2.4	24.2					-46.7	27.9	2.9	7.7
	STOKES	4	6.1 %	4.5	24.5					-60.3	24.8	4.3	7.4
PMC	Plane of maximum curvature												
K	Kyphosis												
L	Lordosis												
°C	Cobb angle												
AV	Apical Vertebra												
Rot	Rotation

**Fig. 5:**
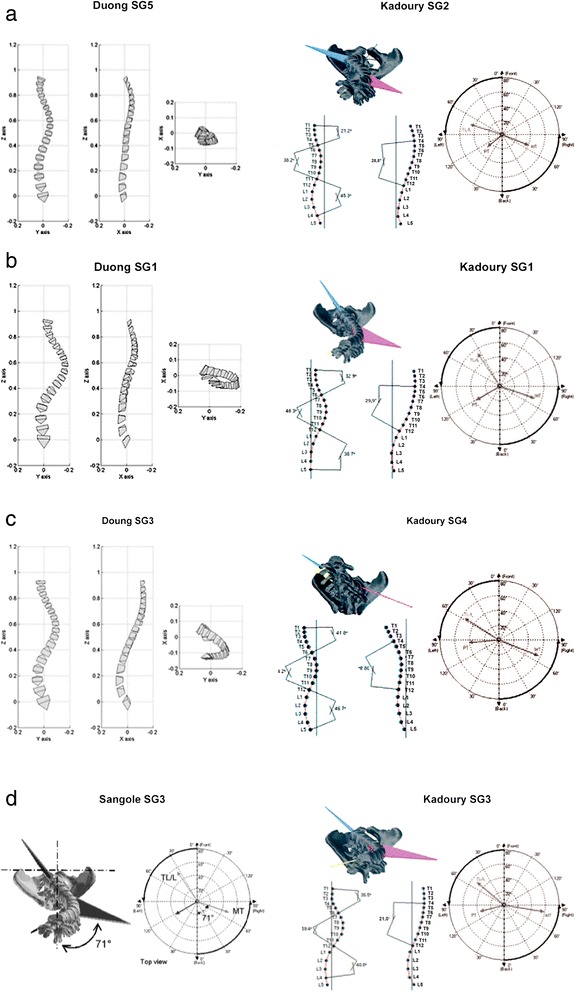
The graphical representations clearly show the similarities among the sub-groups identified by different authors with different methods in different populations of patients. The five 3D Sub-Groups that we found comparing the results of the different authors included: **a** 3D-SG1, important Cobb angle, reduced kyphosis and lordosis; **b** 3D-SG 2, very important Cobb angle, reduced kyphosis and maintained lordosis; **c** 3D-SG3, important Cobb angle, maintained kyphosis and reduced lordosis; **d** 3D-SG4, medium Cobb angle, mild apical rotation and medium PMC rotation; **e** 3D-SG5, important Cobb angle, very low kyphosis, medium apical rotation and high PMC rotation. Images were taken from the papers according to the references: 18; 21–23
- 3D Sub-Group 2, classified as SG1 by Duong and SG1 by Kadoury, characterized by very important Cobb angle (43°–53°), reduced kyphosis (25°–31°) and maintained lordosis (38°–39°). They have been defined by the authors as Class 1 (single thoracic curve pattern similar to a King Type III or a Lenke Type 1 curve, with thoracic hypokyphosis and lumbar hypolordosis in the sagittal plane; the deformity is mainly located in the frontal plane) and Cluster 1 (normal kyphosis with hyper-lordosis and high Cobb angles of the main thoracic curve) (Fig. 5b).
- 3D Sub-Group 3, classified as SG3 by Duong and SG4 by Kadoury, characterized by important Cobb angle (41°–45°), maintained kyphosis (29°–39°) and reduced lordosis (33°–33°). They have been defined by the authors as Class 3 (thoracic and lumbar curve patterns similar to the King I or II, or Lenke Type 3 curves) and Cluster4 (hyper-kyphosis with strong vertebral rotation) (Fig. 5c).
- 3D Sub-Group 4, classified as SG1 by Sangole [18] and SG1 by Stokes [22], characterized by medium Cobb angle (22°–27°), mild apical rotation (6°–5°) and medium PMC rotation (38°–57°). They have been defined by the authors as G1 (smaller, nonsurgical-minor curves) and Group 1 (both curve regions with a plane of maximum curvature rotated counter-clockwise viewed from above) (Fig. 5d).
- 3D Sub-Group 5, classified as SG3 by Sangole [18] and SG3 by Kadoury [21], characterized by important Cobb angle (45°–41°), very low kyphosis (19°–17°), medium apical rotation (16°–11°) and high PMC rotation (45°–90°). They have been defined by the authors as G3 (surgical curves with important PMC rotation and low kyphosis) and Cluster 3 (hypo-kyphosis and hyper-lordosis) (Fig. 5e).

- 3D Sub-Group 1, classified as SG5 by Duong [16, 23] and SG2 by Kadoury [21], characterized by important Cobb angle (42°–39°), reduced kyphosis (23°–26°) and lordosis (30°–32°). They have been defined by the authors as Class 5 (a double thoracic curve similar to a King V or Lenke Type 2 curves) and Cluster 2 (low kyphosis and normal lordosis, with high rotation of PMC) (Fig. 5a).

It was not possible to pair 6 SG out of 16, including two from Stokes with very low degree curves; the others did not have enough comparable data to be matched.

## Discussion

In this paper all the published studies on 3D classifications of scoliosis have been reviewed. It was possible to find 8 papers with different classifications, mostly coming from stereo-radiographies, through clustering techniques or qualitative visual analysis. Two main groups have been identified, one based on the spinal top view, the other on full 3D reconstructions. Even if big differences exist among the studies, some similarities can be found. In addition, among sub-groups identified by single authors there are some similarities that can be linked to usual bi-dimensional radiographic data.

Three-dimensional classification systems for patients with AIS have received an increasing attention because of their importance in assessing the severity and progression of the deformity with the intent of determining optimal surgical and conservative strategies and treatments. Nevertheless, translating complex geometrical concepts into clinically applicable paradigms is complex, therefore, the three – dimensional, quantification and classification of spinal deformities such as AIS remains an open question.

To improve treatment, clinicians should be able to characterize each single peculiar type of curve morphology. Twenty-four vertebral bodies, can combine each other in various manner. The multifaceted manifestations of scoliosis deformities are very well known by experts. Indeed, two markedly different curves may have the same coronal angle. The description of the vertebra kinematics, in terms of spatial position and orientation, needs at least six parameters, considering the characteristic stiffness of each single vertebra. As a consequence, the number of considered vertebras must be multiplied by six to obtain the minimum number of parameters able to describe the spine. This complex model cannot be adopted to directly produce an analytical quantitative clinical picture of each single patient. Therefore, a classification system is needed, it should be able to group subjects, by summing up this huge number of parameters. These classification models are based on arbitrary choices made by the researcher who decide to focus on specific clinical aspects. To make the best choices, researchers will need clinically validated parameters and comparisons between different classification systems. Some top view parameters seem to represents the ideal parameter able to globally define the characteristics of different scoliosis pattern [17, 19, 20]. Moving from a 2D to a 3D classification will help mainly for two crucial aspects: first of all, it will help to perform a more precise prognosis for each single patient [26]. Secondly, it will help clinicians in applying a more effective treatment [30]. Bracing is becoming more and more complex: after being focused on the elongation when the Milwaukee brace was first developed, the studies moved to a 3 point system like the Lyon brace. In more recent years, braces became really three dimensional, adding a detorsion action and considering the whole shape of the trunk and its deformity [31, 32]. The main expression of these new evolutions are the Rigo Cheneau system [33, 34], the PASB brace [35] the Sforzesco brace [36] from which new braces were developed [37]. The Physioterapic Specific Scoliosis Exercise (PSSE), had a similar evolution, with the most up to date protocols applying a three dimensional active self correction [38]. Unfortunately, for both treatments, braces and exercise, clinicians must rely mainly on their own experience than on an objective 3D classification.

Recent studies presented in this review have investigated and proposed new classification systems of spine deformities based on explicit 3D geometrical descriptors of the spine. These proposals of 3D classification neither have gained wide acceptance nor are they used in clinical practice. Several reasons can be proposed:

- The inherent complexity in interpretation associated with some methods of measurements and classification parameters: they are not intuitive for clinicians for clinical everyday activity, and cannot be easily related to usual curve pattern identification on radiographs.
- 3D reconstructions of the spine are not very simple and the equipment necessary to obtain reconstructions in the standing position is not readily available to a majority of clinicians: in fact they have been used up to now mostly in a research context. Recent developments now allow the possibility to obtain fast and minimally irradiating 3D reconstructions of the spine [39–41].
- It has to be shown that these classifications are clinically relevant and valid.
- Among clinicians there may be people not ready to change their clinical modalities with new complex, expensive technologies.

In this respect, when looking at a new scale and/or classification, there are several requirements that must be fulfilled. A valid classification system [20]:

- Should evaluate and characterize the 3-D of the spine using shape indices to describe it.
- Must be feasible and useful in real everyday clinical practice as a guide for appropriate patient management and should help in treatment decisions demonstrating the possibility of future applications in everyday settings with usual clinical instruments.
- Must be comparable to other 3-D classifications, to find out the easiest and most reliable one.
- Must produce something different from 2-D classifications, but anyway inherent to 3-D deformities.
- Should predict clinical results.
- Should be complete and cover the wide variety of curve patterns.
- Should be generalizable in other settings.
- Should be feasible, quick and easy to use.

There are some advantages coming from the automatic collection of parameters offered by all these new technologies: they are able to offer to clinicians a larger amount of standardized data from long term follow up, with a significant time saving. Until now all these new technologies, have not been tested for reliability of measurements, therefore further studies are needed.

These novel technologies will require long training periods for the professionals involved, to avoid any kind of operators’ measurement errors.

The main limit of the present review is the lack of comparison among the different classification found. The heterogeneity of works and, in particular, of the instruments used, of the 3D analysis methods, as differences in classificatory parameters, classification methodology and results obtained have prevented any kind of systematic comparison, nor a true metanalysis.

Future evolutions could include:

- Selection of the appropriate key features eventually by consensus among expert spine clinicians, surgeons and bioengineers.
- The best classification is the most reliable with the best repeatability: so we need repeatability and reliability tests for each classification, if not yet done.
- Comparison studies of the different classification, by choosing the most similar: this will imply a different classification process in the same sample of scoliosis patients, in long term follow up.
- Modelling and regression analysis to verify the predictive value of different parameters.
- Reliability test of the new technologies for automatic evaluations.
- The implementation of the currently available new technologies in clinical everyday practice, by making them more accessible.

In summation, the state of the art of 3D classification systems include 8 studies of almost 1200 patients. Studies have some comparability, even though of low level. Therefore, the most useful one in clinical everyday practice, is far from being defined. Nevertheless, after more than 20 years from the definition of the importance of the third dimension of the deformity, the time has come for clinicians and bioengineers to start some real clinical application, and develop means to make this approach an everyday tool.
